# Machine Learning–Driven Models to Predict Prognostic Outcomes in Patients Hospitalized With Heart Failure Using Electronic Health Records: Retrospective Study

**DOI:** 10.2196/24996

**Published:** 2021-04-19

**Authors:** Haichen Lv, Xiaolei Yang, Bingyi Wang, Shaobo Wang, Xiaoyan Du, Qian Tan, Zhujing Hao, Ying Liu, Jun Yan, Yunlong Xia

**Affiliations:** 1 Department of Cardiology The First Affiliated Hospital of Dalian Medical University Dalian China; 2 Medical Department Yidu Cloud (Beijing) Technology Co Ltd Beijing China; 3 College of Life Science and Bioengineering Beijing University of Technology Beijing China; 4 AI Lab Yidu Cloud (Beijing) Technology Co Ltd Beijing China; 5 Medical Department Happy Life Technology Co Ltd Beijing China

**Keywords:** heart failure, machine learning, predictive modeling, mortality, positive inotropic agents, readmission

## Abstract

**Background:**

With the prevalence of cardiovascular diseases increasing worldwide, early prediction and accurate assessment of heart failure (HF) risk are crucial to meet the clinical demand.

**Objective:**

Our study objective was to develop machine learning (ML) models based on real-world electronic health records to predict 1-year in-hospital mortality, use of positive inotropic agents, and 1-year all-cause readmission rate.

**Methods:**

For this single-center study, we recruited patients with newly diagnosed HF hospitalized between December 2010 and August 2018 at the First Affiliated Hospital of Dalian Medical University (Liaoning Province, China). The models were constructed for a population set (90:10 split of data set into training and test sets) using 79 variables during the first hospitalization. Logistic regression, support vector machine, artificial neural network, random forest, and extreme gradient boosting models were investigated for outcome predictions.

**Results:**

Of the 13,602 patients with HF enrolled in the study, 537 (3.95%) died within 1 year and 2779 patients (20.43%) had a history of use of positive inotropic agents. ML algorithms improved the performance of predictive models for 1-year in-hospital mortality (areas under the curve [AUCs] 0.92-1.00), use of positive inotropic medication (AUCs 0.85-0.96), and 1-year readmission rates (AUCs 0.63-0.96). A decision tree of mortality risk was created and stratified by single variables at levels of high-sensitivity cardiac troponin I (<0.068 μg/L), followed by percentage of lymphocytes (<14.688%) and neutrophil count (4.870×10^9^/L).

**Conclusions:**

ML techniques based on a large scale of clinical variables can improve outcome predictions for patients with HF. The mortality decision tree may contribute to guiding better clinical risk assessment and decision making.

## Introduction

Heart failure (HF) syndrome is a life-threatening chronic disorder with a global prevalence that has been rising consistently over recent decades because of population aging, shifts in disease spectrums, and improved survival rates among patients with various cardiovascular diseases [1,2]. HF is characterized by complex therapeutic regimens, frequent hospitalizations, and a poor prognosis, resulting in a tremendous health care burden [3]. In certain instances, these issues are also fundamental targets of a strategy for HF prevention and treatment [4,5]. It is crucial to discriminate accurately among patients with HF to identify those who have a high risk of in-hospital mortality and readmission, as well as to guide the use of different therapies based on patients' features.

A prediction model was developed as an important risk assessment tool and used in various health care areas over the past decades. It has been recognized to facilitate early identification of patients at disease or event risk and enables effective interventions for those who might benefit most from identifying specific risk factors [6,7]. Previous studies in the field of cardiology, generally based on different populations with HF, have constructed models that are relevant to prognosis prediction, including the Seattle Heart Failure Model (SHFM) [8], Munich score [9], Enhanced Feedback for Effective Cardiac Treatment (EFFECT) scale [10], and Acute Decompensated Heart Failure National Registry (ADHERE) [11]. Moreover, several available parameters related to increased mortality have been identified as well, such as age [10,12,13], concentration of B-type natriuretic peptide (BNP) [14,15], urea nitrogen level [10,12], and systolic blood pressure (SBP) [9,13]. Although the model’s construction from such cohorts or databases provides a level of concrete evidence, it is typically limited to large volumes of clinical resources and unstructured data [16]. Given the growing popularity of big data use and mining, data derived from electronic health records (EHRs) are becoming more available and accessible for clinical research.

Besides this, predictive models have been constructed in various health care domains with a certain degree of success by automated mining of EHRs, combined with machine learning (ML) approaches [17], specifically for the prediction of HF outcomes [18-20]. In contrast to previous studies of predictive models for HF outcomes that apply traditional methods, recent research is adopting ML techniques for predicting HF mortality, readmission, and medication adherence, which might demonstrate better performance in their predictions because of their consideration of higher order and nonlinear relationships between multidimensional variables [21].

In our study, we explored the use of the traditional method—logistic regression (LR)—and the four novel ML approaches—support vector machine (SVM), artificial neural network (ANN), random forest (RF), and extreme gradient boosting (XGBoost)—to predict prognostic outcomes for subjects with HF in real-world settings. We demonstrated the development of EHR-based models to predict the 1-year in-hospital mortality, use of positive inotropic agents, and all-cause readmissions in a single year.

## Methods

### Patients and Data Source

We collected the EHR data from hospitalized patients diagnosed with HF at the First Affiliated Hospital of Dalian Medical University (Liaoning Province, China) over 7 years between December 2010 and August 2018. All patients with HF were diagnosed and treated according to institutional guidelines. Because the diagnostic terminologies of EHRs have been structured and normalized according to the International Statistical Classification of Diseases and Related Health Problems, 10th Revision (ICD-10 [22]), newly diagnosed patients with HF (aged >18 years) were screened by a dynamically updated big data intelligence platform developed by an artificial intelligence technology company in collaboration with hospitals (Yidu Cloud Technology Co., Ltd). Each patient’s data were extracted from various EHR systems integrated into a single platform, including the hospital information system, electronic medical record, radiation information system, laboratory information system, ultrasound system, and electrocardiogram system. The study was finally approved by the ethics committee of the First Affiliated Hospital of Dalian Medical University. Written informed consent was waived due to the retrospective design.

### Selection of Variables

The platform incorporates comprehensive and detailed data on patients’ routine health care. Common and cardiovascular-specific variables were structured and normalized by natural language processing, ML techniques, and well-defined logical rules. Considering the large scale of EHR data and the advantages of ML techniques, candidate variables with known clinical significance and inaccessible through traditional medical records were collected in our study. We excluded variables with missing values greater than 20%. Finally, a total of 79 variables related to the first hospitalization were extracted from a big data intelligence platform. The features were as follows: demographics (age and sex), personal history (smoking and drinking), history (comorbidities and surgery), etiology, vital signs, routine laboratory examinations, interventions, and medication use on admission (Multimedia Appendix 1). Repeated measurements of vital signs and laboratory tests from patients with HF were taken over different periods to ensure data accuracy.

### Outcomes

We established models using 5 algorithms separately to predict the primary outcome: all-cause in-hospital mortality within 1 year. The secondary outcomes were (1) use of positive inotropic agents for patients with HF and (2) all-cause readmission. Mortality was defined as a clear record of death of an inpatient within 1 year of hospitalization. The following commonly used positive inotropic agents in clinical practice were selected for our study: dopamine, dobutamine, milrinone, levosimendan, and deslanoside. Readmission was defined as any patient with an interval of more than 1 day from the last discharge until the next admission; no readmitted patients died within 1 year of hospitalization. Therefore, enrolled patients were labeled as “deceased” or “survivors.”

### ML Model Development and Performance Evaluation

The synthetic minority oversampling technique was first employed to address the imbalance in the data set, with a ratio of 1:1 between deceased patients and survivors. It is common for a minority to be oversampled by deriving new, “synthetic” samples to alleviate imbalance [23].

The hold-out method was used to divide the data set into a training/validation data set (90%) and a hold-out test set (10%). Five approaches were explored with 10-fold cross-validation for building models and adjusting parameters: LR, RF, SVM, ANN, and XGBoost. The hold-out test set was used to evaluate the best-performing models created with the training set. The area under the curve (AUC) of the receiver operating characteristic (ROC) curve was chosen as the primary evaluation metric for our models, including accuracy, precision, and recall. The Brier score (range of 0 to 1)—the average squared error between the predicted and the actual value—was also commonly represented as a “calibration” for overall measurement. Shapley additive explanation (SHAP) values were used to evaluate feature importance.

### LR Method

LR is the most basic dichotomous linear method of model selection that makes classification decisions. LR is superior in measuring the probability between “0” and “1” based on the relationships of binary classifications in continuous or categorical variables [24]. We used the sklearn.linear module to develop the LR models.

### RF Method

RF is an algorithm that integrates multiple decision tree classifiers. Each node of the decision tree represents a predictive variable to separate the outcome classes by setting the optimal threshold. The importance of features can also be obtained with the sum of weights of the classifier’s nodes [25]. The RF method is generally used to deal with thousands of input variables without dimension reduction. We used the RF classifier from the sklearn.ensemble module to develop the models. The related parameters (n_estimators, max_features, max_depth, and min_samples_split) were adjusted to prevent poor results and overfitting.

### SVM Method

As a dichotomous supervised algorithm, SVM can be used in high-dimensional feature space. The best hyperplane can be achieved using a kernel-based function for separating two classes at maximum intervals [26].

### ANN Method

A multilayer perceptron (MLP) classifier was implemented using the sklearn.neural_network module to develop models [27]. MLP is a popular ANN method that generally consists of neurons from the input, hidden, and output layers. The data were processed through weighted connections and activation functions in the hidden layers [28]. In our study, the two hidden layers had 40 and 20 neurons. The rectified linear unit was chosen as their activation function.

### XGBoost Method

XGBoost is one of the boosting methods; this algorithm aims to integrate weak classifiers into a single robust classifier in an iterative fashion [29]. This algorithm constructs a scalable classification and regression tree in a boosting ensemble manner on a gradient boosting decision tree basis, which can learn nonlinear relationships among variables and outcomes flexibly and accurately [30]. The small learning rate (0.1) indicates better generalization. The tree number and maximum depth were limited to 80 (estimators) and 3, respectively.

### Statistical Analysis

All ML algorithms were performed using the scikit-learn (version 0.21.1) package in Python (version 3.6.5; Python Software Foundation), and statistical analysis was conducted using an open-source Scipy (version 1.3.0) database from Python (version 3.6.3). All categorical data were presented as percentages. All continuous data performing a normal distribution were presented as mean (SD); otherwise, they were expressed as median (IQR). Student *t* tests or chi-square tests were applied for group comparisons. *P* values <.05 were considered statistically significant.

## Results

### Baseline Characteristics

A total of 13,602 hospitalized patients with newly diagnosed HF were enrolled in this study, of whom 3.95% (n=537) died in hospital within 1 year. The usage rate of positive inotropic agents was 20.43% (n=2779). The overall all-cause readmission rates of 30 days, 60 days, and 1 year for patients with HF were 4.83%, 14.77%, and 21.16%, respectively (n=657, n=2009, and n=2878, respectively, of 13,602 cases). The eligible population’s baseline characteristics were compared between 2 groups according to survival status (Table 1). Patients in the deceased group were older than those in the survivor group (77 years, IQR 66.5-83.0 years versus 72 years, IQR 63.0-80.0 years, respectively). The proportion of male patients in both groups was 52.5% (282/537 and 6860/13,065 in the deceased group and survivor group, respectively). The number of patients with comorbid diagnoses of diabetes, hypertension, and tumors was remarkably different between the 2 groups (all *P*<.001). Concerning the etiology of HF, the number of patients with cardiomyopathy and cardiac arrhythmia was found to be different between the deceased and survivor groups (both *P*<.001). There was a significant difference in the vital signs (heart rate and blood pressure) of patients with HF between the 2 groups (all *P*<.001). In-hospital medication use (angiotensin-converting enzyme inhibitors [ACEIs] and aldosterone receptor antagonists [ARBs]) was higher among subjects who survived in the hospital (*P*<.001).

**Table 1:** Demographic and clinical variables of the deceased and survivor groups (N=13,602).

Variables					Deceased group			Survivor group			*P* value
Total patients, n (%)					537 (3.9)			13,065 (96.1)			
**Demographic information**											
	Age (years), median (IQR)			77.0 (66.5-83.0)			72.0 (63.0-80.0)			.003	
	Gender (male), n (%)			282 (52.5)			6860 (52.5)			.997	
	Smoking history, n (%)			115 (21.4)			3282 (25.1)			.05	
	Drinking history, n (%)			64 (11.9)			1820 (13.9)			.19	
**Comorbidities, n (%)**											
	Diabetes mellitus			202 (37.6)			3593 (27.5)			<.001	
	Hypertension			338 (62.9)			7073 (54.1)			<.001	
	Dyslipidemia			313 (58.2)			8449 (64.7)			.003	
	COPD^a^			3 (0.5)			50 (0.4)			.47	
	Chronic renal disease			13 (2.4)			144 (1.1)			.005	
	Tumors			46 (8.6)			534 (4.1)			<.001	
**Etiology of heart failure, n (%)**											
	Coronary heart disease			349 (65.0)			7810 (59.8)			.02	
	Cardiomyopathy			22 (4.1)			1207 (9.2)			<.001	
	Valvular heart disease			91 (16.9)			2389 (18.3)			.43	
	Cardiac arrhythmia			193 (35.9)			5780 (44.2)			<.001	
	History of cardiovascular surgery			115 (21.4)			2519 (19.3)			.22	
**Vital signs, median (IQR)**											
	**Blood pressure (mmHg)**										
		Diastolic	77.0 (68.0-84.0)			80.0 (70.0-90.0)			<.001		
		Systolic	130.0 (115.0-150.0)			140.0 (120.0-152.0)			<.001		
	Heart rate (beats/min)			84.0 (72.0-99.0)			76.0 (68.0-90.0)			<.001	
	Respiratory rate (breaths/min)			19.0 (18.0-20.0)			18.0 (17.0-19.0)			.66	
	Temperature			36.2 (36.0-36.5)			36.2 (36.0-36.4)			.003	
**NYHA^b^ classification, n (%)**											<.001
	IV			113 (21.0)			1424 (10.9)				
	III			96 (17.9)			4006 (30.7)				
	II			22 (4.1)			1738 (13.3)				
	I			0 (0)			12 (0.1)				
	None			297 (55.3)			5291 (40.5)				
**Laboratory indicators at admission, median (IQR)**											
	BNP^c^			1053.5 (399.5-2383.3)			322.9 (106.6-845.0)			<.001	
	hs-cTnl^d^			0.4 (0.1-5.2)			0.03 (0.01-0.11)			<.001	
	Creatine kinase MB (U/L)			2.8 (1.4-8.0)			1.5 (0.8-2.6)			<.001	
	Hemoglobin (g/L)			115.0 (94.0-133.0)			131.0 (117.0-144.0)			<.001	
	Platelets			180.5 (125.8-242.0)			193.0 (155.0-235.0)			.001	
	White blood cells (×10^9^/L)			9.5 (6.4-14.0)			6.6 (5.3-8.2)			<.001	
	Red blood cells			3.9 (3.2-4.4)			4.3 (3.9-4.8)			<.001	
	Lymphocytes			1.1 (0.7-1.7)			1.6 (1.1-2.1)			.59	
	Neutrophils			7.1 (4.5-11.3)			4.1 (3.1-5.5)			<.001	
	Mean platelet volume (fL)			10.8 (10.0-11.7)			10.7 (10.0-11.4)			.004	
	Hematocrit			34.1 (26.1-39.7)			38.7 (33.2-42.7)			<.001	
	Basophils (×10^9^/L)			0.02 (0.01-0.03)			0.02 (0.01-0.04)			.55	
	Monocytes (×10^9^/L)			0.6 (0.4-0.9)			0.5 (0.4-0.7)			<.001	
	Monocytes (%)			6.7 (4.5-9.0)			7.9 (6.4-9.7)			<.001	
	Mean corpuscular volume (fL)			91.1 (87.8-94.2)			91.0 (87.9-94.2)			.62	
	Procalcitonin			0.4 (0.1-1.9)			0.1 (0.1-0.3)			.20	
	Neutrophils (%)			78.3 (67.6-87.2)			63.1 (55.5-71.1)			<.001	
	Basophils (%)			0.2 (0.1-0.4)			0.4 (0.2-0.5)			<.001	
	Eosinophils (%)			0.8 (0.2-1.9)			1.7 (0.9-2.9)			<.001	
	Eosinophils (×10^9^/L)			0.1 (0.03-0.2)			0.1 (0.1-0.2)			.002	
	Lymphocytes (%)			12.7 (6.7-21.4)			25.6 (18.2-32.7)			<.001	
	Total bilirubin (µmol/L)			16.3 (11.2-27.3)			14.5 (10.5-20.5)			<.001	
	Direct bilirubin (µmol/L)			5.4 (3.4-9.4)			4.6 (3.2-6.9)			<.001	
	Glucose (mmol/L)			6.5 (5.1-9.3)			5.5 (4.9-6.8)			<.001	
	Lipoprotein(a) (mg/L)			165.3 (84.7-307.5)			152.1 (83.0-277.0)			.08	
	High-density lipoprotein cholesterol (mmol/L)			1.3 (0.9-34.0)			1.5 (1.0-39.0)			.01	
	Low-density lipoprotein cholesterol (mmol/L)			3.2 (2.2-80.0)			3.5 (2.3-90.0)			.08	
	Total cholesterol (mmol/L)			5.3 (3.9-137.3)			5.8 (4.2-155.0)			.03	
	Triglycerides (mmol/L)			1.8 (1.0-80.0)			2.1 (1.1-91.0)			.01	
	Alanine aminotransferase (U/L)			27.0 (14.0-61.0)			20.0 (13.0-33.0)			<.001	
	Aspartate aminotransferase (U/L)			36.0 (20.0-101.0)			21.0 (16.0-30.0)			<.001	
	Gamma-glutamyl transferase (U/L)			46.0 (25.0-87.0)			35.0 (22.0-63.0)			<.001	
	Albumin (g/L)			35.0 (30.6-38.6)			39.3 (36.2-41.9)			<.001	
	Globulin (g/L)			28.6 (24.4-32.9)			26.7 (23.6-30.3)			<.001	
	Albumin/globulin ratio			1.2 (1.0-1.5)			1.5 (1.3-1.7)			<.001	
	Total protein (g/L)			63.1 (58.1-69.3)			65.9 (61.5-70.4)			<.001	
	Creatinine (μmol/L)			110.0 (77.0-201.0)			77.0 (62.0-98.0)			<.001	
	Sodium (mmol/L)			138.5 (135.0-142.0)			141.0 (138.8-143.0)			<.001	
	Potassium (mmol/L)			4.0 (3.7-4.5)			4.0 (3.7-4.3)			<.001	
	Calcium (mmol/L)			2.1 (2.0-2.2)			2.2 (2.1-2.3)			<.001	
	Uric acid (μmol/L)			446.0 (321.8-611.3)			390.0 (311.0-489.0)			<.001	
	Urea (mmol/L)			11.4 (7.4-19.3)			7.1 (5.6-9.5)			<.001	
	Alkaline phosphatase (U/L)			84.0 (69.0-116.0)			74.0 (62.0-92.0)			<.001	
	Acetylcholinesterase (U/L)			210.5 (148.0-281.0)			292.0 (229.0-363.0)			<.001	
	International normalized ratio			1.2 (1.1-1.4)			1.1 (1.0-1.21)			<.001	
	Prothrombin time (s)			13.2 (11.9-15.8)			11.9 (11.0-13.2)			<.001	
	Fasting blood glucose (g/L)			3.6 (2.7-4.5)			3.0 (2.5-3.7)			<.001	
	Activated partial thromboplastin time (s)			31.7 (26.5-41.8)			26.8 (23.9-30.8)			<.001	
**Use of devices during hospitalization, n (%)**											
	Cardiac resynchronization therapy			2 (0.4)			42 (0.3)			.69	
	ICD^e^ implantation			0 (0)			32 (0.2)			.64	
	Permanent pacemaker			3 (0.6)			350 (2.7)			.002	
	Temporary pacemaker			0 (0)			15 (0.1)			>.99	
**Medication use during hospitalization, n (%)**											
	ACEI^f^/ARB^g^			207 (38.5)			7967 (61.0)			<.001	
	β-blocker			408 (76.0)			10,199 (78.1)			.25	
	Aldosterone antagonist			266 (49.5)			8364 (64.0)			<.001	
	Statin			310 (57.7)			8399 (64.3)			.002	
	Aspirin			331 (61.6)			8255 (63.2)			.47	
	Diuretic			508 (94.6)			10,927 (83.6)			<.001	
	Digoxin			73 (13.6)			2424 (18.6)			.004	
**Outcome, n (%)**											
	**Positive inotropic agents use**										
		Dopamine	319 (59.4)			1407 (10.8)			<.001		
		Dobutamine hydrochloride	39 (7.3)			225 (1.7)			<.001		
		Milrinone	52 (9.7)			325 (2.5)			<.001		
		Levosimendan	7 (1.3)			37 (0.3)			.002		
		Lanatoside C	129 (24.0)			1132 (8.7)			<.001		
	**Readmissions**										
		30 days	53 (9.9)			604 (4.6)			<.001		
		180 days	129 (24.0)			1880 (14.4)			<.001		
		1 year	162 (30.2)			2716 (20.8)			<.001		

^a^COPD: chronic obstructive pulmonary disease.

^b^NYHA: New York Heart Association.

^c^BNP: B-type natriuretic peptide.

^d^hs-cTnI: high-sensitivity cardiac troponin I.

^e^ICD: implantable cardioverter defibrillator.

^f^ACEI: angiotensin-converting enzyme inhibitor.

^g^ARB: angiotensin receptor blocker.

### 1-Year In-Hospital Mortality Models

**Figure 1:**
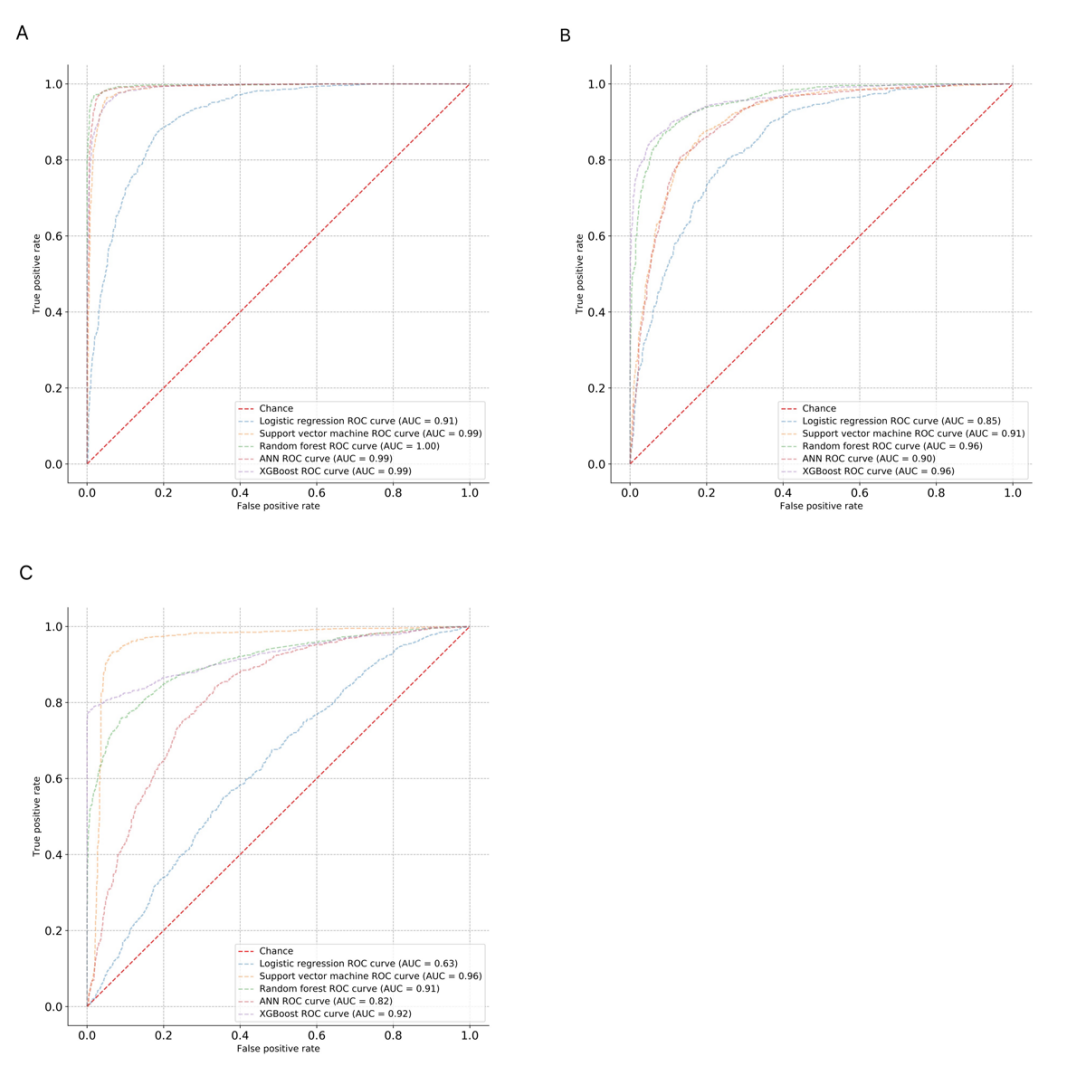
Receiver operating characteristic (ROC) curves using the synthetic minority oversampling technique for the logistic regression, random forest, support vector machine, artificial neural network (ANN), and extreme gradient boosting (XGBoost) models in predicting (A) 1-year in-hospital mortality, (B) use of positive inotropic agents, and (C) 1-year all-cause readmission. AUC: area under the curve.

**Figure 2:**
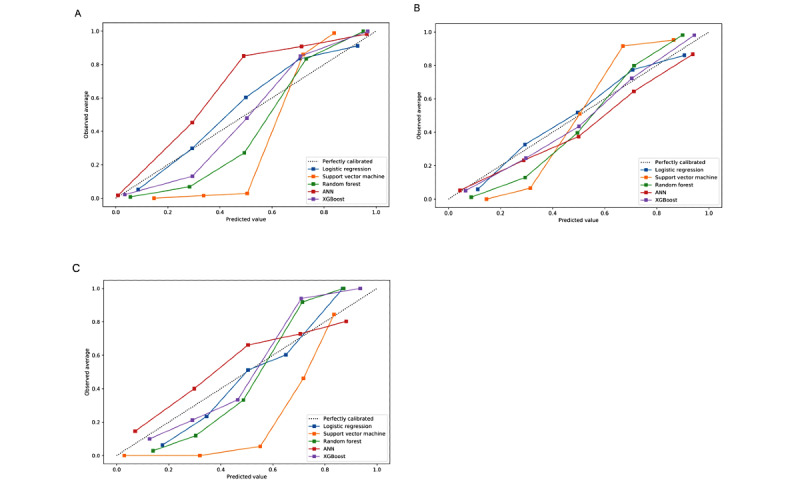
Calibration plots using the synthetic minority oversampling technique for the logistic regression, random forest, support vector machine, artificial neural network (ANN), and extreme gradient boosting (XGBoost) models in predicting (A) 1-year in-hospital mortality, (B) use of positive inotropic agents, and (C) 1-year all-cause readmission.

Predictive models for 1-year in-hospital mortality risk assessment were conducted using 5 algorithms. Figure 1A shows the performances of models in the form of AUC. The AUC values for LR, RF, SVM, ANN, and XGBoost were 0.91, 1.00, 0.99, 0.99, and 0.99, respectively. RF had relatively higher AUC than the other algorithms. The calibration plots of our 5 methods are presented in Figure 2A. Four ML models had an accuracy of higher than 95%. Regarding precision, the RF and ANN algorithms emerged as the best, achieving the highest precision (0.96), followed by the SVM (0.93) and XGBoost (0.91) algorithms. The Brier score for RF and ANN was the lowest (0.03) (Table 2).

**Table 2:** Performance of the machine learning approaches for the estimation of 1-year in-hospital all-cause mortality.

Model	AUC^a^	Accuracy	Precision	Recall	F1	Brier score
LR^b^	0.91	0.83	0.86	0.80	0.83	0.12
RF^c^	1.00	0.97	0.96	0.98	0.97	0.03
SVM^d^	0.99	0.94	0.93	0.96	0.94	0.16
ANN^e^	0.99	0.97	0.96	0.98	0.97	0.03
XGBoost^f^	0.99	0.94	0.91	0.98	0.94	0.05

^a^AUC: area under the curve.

^b^LR: logistic regression.

^c^RF: random forest.

^d^SVM: support vector machine.

^e^ANN: artificial neural network.

^f^XGBoost: extreme gradient boosting.

**Figure 3:**
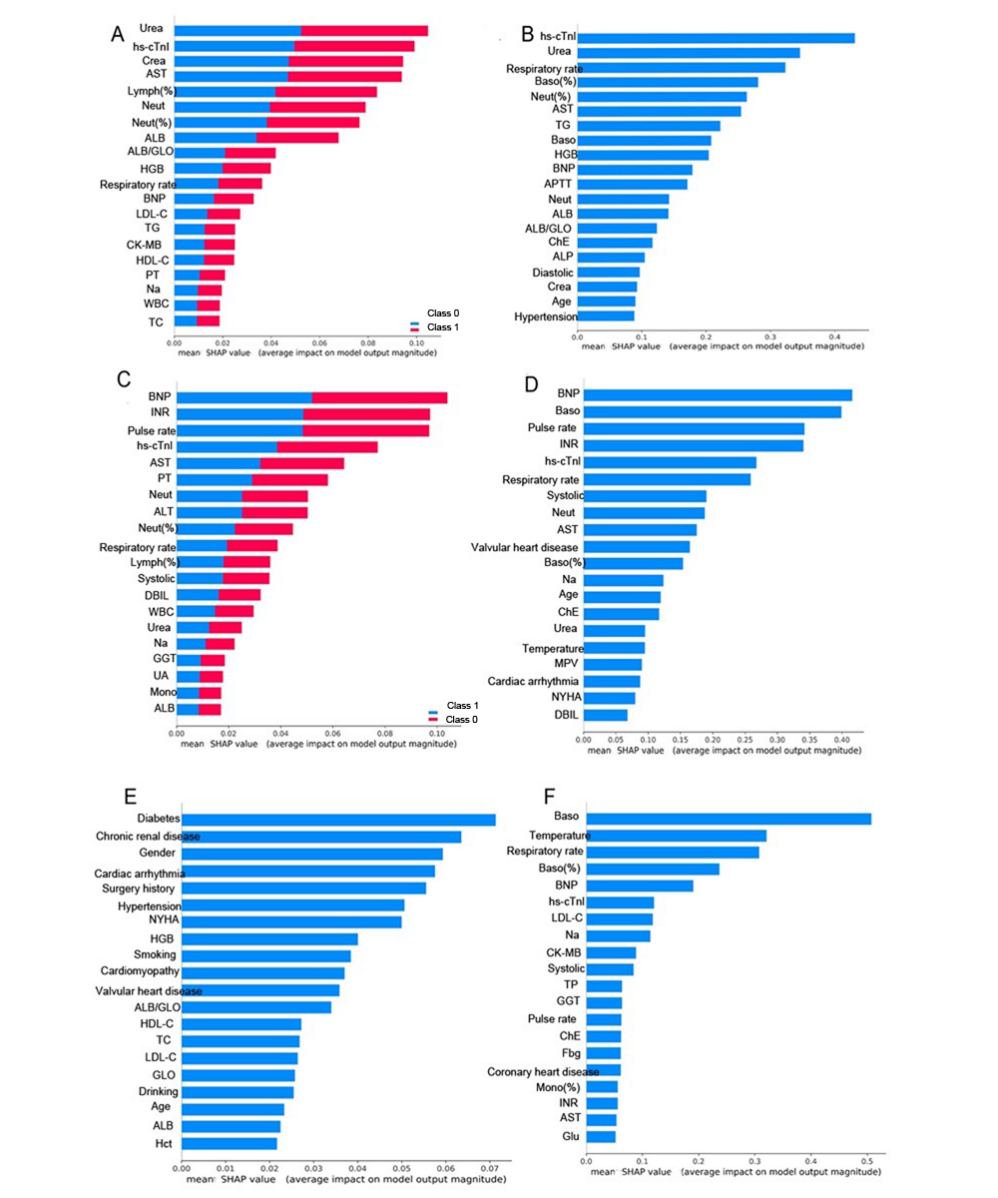
Shapley additive explanation (SHAP) plots for the machine learning models in predicting (A) 1-year in-hospital mortality using the random forest (RF) method, (B) 1-year in-hospital mortality using the extreme gradient boosting (XGBoost) method, (C) use of positive inotropic agents using the RF method, (D) use of positive inotropic agents using the XGBoost method, (E) 1-year all-cause readmission using the support vector machine (SVM) method, and (F) 1-year all-cause readmission using the XGBoost method. ALB: albumin; ALP: alkaline phosphatase; APTT: activated partial thromboplastin time; AST: aspartate aminotransferase; Baso: basophils; BNP: B-type natriuretic peptide; ChE: cholinesterase: CK-MB: creatine kinase MB; COPD: chronic obstructive pulmonary disease; Crea: creatinine; DBIL: direct bilirubin; Fbg: fibrinogen; GGT: gamma-glutamyl transferase; GLO: globulin; Glu: glucose; Hct: hematocrit; HDL-C: high-density lipoprotein cholesterol; HGB: hemoglobin; hs-cTnI: high-sensitivity cardiac troponin I; INR: international normalized ratio; LDL-C: low-density lipoprotein cholesterol; Lymph(%): percentage of lymphocytes; Mono: monocytes; MPV: mean platelet volume; Na: sodium; Neut: neutrophils; NYHA: New York Heart Association; PT: prothrombin time; TC: total cholesterol; Systolic: systolic blood pressure; TG: triglycerides; TP: total protein; UA: uric acid; WBC: white blood cells.

Furthermore, we explored the importance of the features that affect mortality prediction by applying RF and XGBoost approaches (Figure 3), with the weight assignment of each feature expressed as a SHA*P* value based on whether it favored judgment in survival. As shown in Figure 3A and 3B, blood urea, high-sensitivity cardiac troponin I (hs-cTnI), creatinine, aspartate aminotransferase (AST), and percentage of lymphocytes were the top 5 related variables in mortality prediction. In contrast, hs-cTnI was the most crucial marker identified using the XGBoost algorithm, followed by urea, respiration rate, percentage of basophils, and percentage of neutrophils.

### Positive Inotropic Agent Use Models

Figure 1B demonstrates the ROC of the predictive models in patients' use of positive inotropic agents. In comparing AUCs among the 5 models, the XGBoost and RF models had the highest AUC value (0.96), followed by the SVM model (0.91), ANN model (0.90), and LR model (0.85). In consideration of precision (0.85) and recall (0.91) values, RF was determined to be the best method for prediction (Table 3). As shown in calibration curves (Figure 2B), a nonparametric plot of the RF algorithm was close along the ideal diagonal line and had the lowest Brier score (0.10).

**Table 3:** Performance of the machine learning approaches for the estimation of use of positive inotropic agents.

Model	AUC^a^	Accuracy	Precision	Recall	F1	Brier score
LR^b^	0.85	0.78	0.77	0.79	0.78	0.16
RF^c^	0.96	0.87	0.85	0.91	0.88	0.10
SVM^d^	0.91	0.85	0.83	0.88	0.84	0.17
ANN^e^	0.90	0.83	0.78	0.94	0.84	0.12
XGBoost^f^	0.96	0.84	0.79	0.94	0.86	0.11

^a^AUC: area under the curve.

^b^LR: logistic regression.

^c^RF: random forest.

^d^SVM: support vector machine.

^e^ANN: artificial neural network.

^f^XGBoost: extreme gradient boosting.

Interestingly, BNP, international normalized ratio (INR), pulse rate, hs-cTnI, and AST were the top 5 markers predicting use of positive inotropic agents by the RF approach. BNP was also identified as a critical marker for forecasting positive inotropic agent use in the XGBoost model, followed by basophil counts, pulse rate, INR, and hs-cTnI (Figure 3C and 3D).

### 1-Year All-Cause Readmission Models

The discrimination of different models for 1-year all-cause readmissions represented by AUCs is shown in Figure 1C. The SVM method achieved the best performance in terms of 1-year readmission prediction (AUC 0.96). XGBoost showed the lowest Brier score for calibration plots (0.12), followed by RF (0.13), SVM (0.16), ANN (0.18), and LR (0.24) (Figure 2C and Table 4).

**Table 4:** Performance of the machine learning approaches for the estimation of 1-year all-cause readmission.

Model	AUC^a^	Accuracy	Precision	Recall	F1	Brier score
LR^b^	0.63	0.57	0.57	0.59	0.58	0.24
RF^c^	0.91	0.82	0.83	0.81	0.82	0.13
SVM^d^	0.96	0.90	0.86	0.96	0.91	0.16
ANN^e^	0.82	0.74	0.74	0.75	0.74	0.18
XGBoost^f^	0.92	0.83	0.82	0.84	0.83	0.12

^a^AUC: area under the curve.

^b^LR: logistic regression.

^c^RF: random forest.

^d^SVM: support vector machine.

^e^ANN: artificial neural network.

^f^XGBoost: extreme gradient boosting.

Of the 79 variables analyzed, the presence of diabetes was identified as the most important marker for prediction of 1-year all-cause readmission using the SVM method, whereas basophil count was a significant predictor of readmission using the XGBoost algorithm. There appeared to be a certain discrepancy in the ranking of other features derived by the two methods (Figure 3E and 3F).

### Mortality Risk Assessment Model

**Figure 4:**
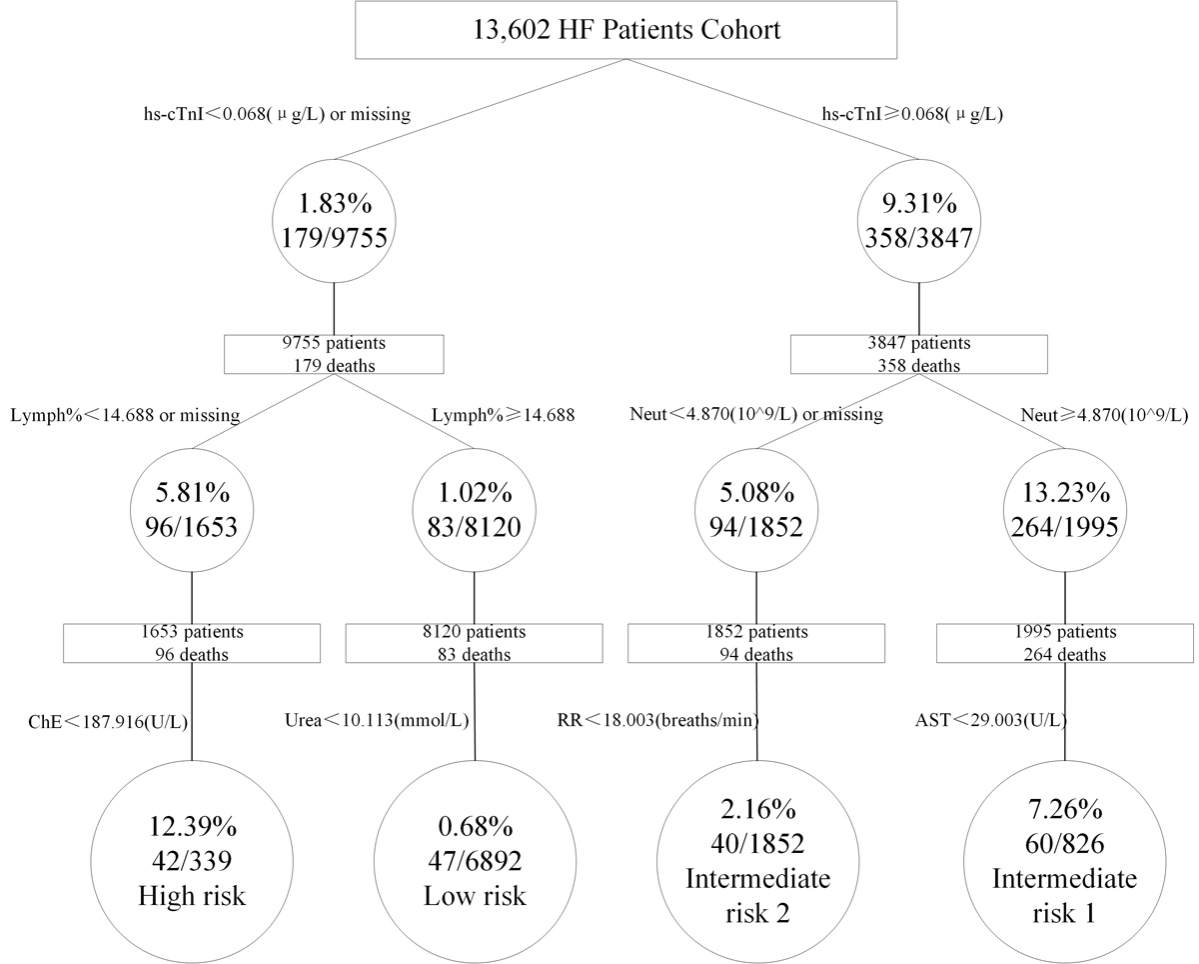
Predictors of 1-year in-hospital mortality and risk stratification using an extreme gradient boosting (XGBoost) algorithm. AST: aspartate transaminase; ChE: cholinesterase; HF: heart failure; hs-cTnI: high-sensitivity cardiac troponin I; Lymph%: percentage of lymphocytes; neut: neutrophil count; RR: respiration rate.

The patients were divided into subgroups of high-, intermediate-, and low-risk in-hospital mortality according to the cutoff value of various variables obtained using the XGBoost algorithm (Figure 4). Of the 79 variables, the hs-cTnI level (<0.068 μg/L) was identified as the first single predictor to discriminate between deceased and surviving patients. The next discriminator in the left node of hs-cTnI was the percentage of lymphocytes at a discrimination level of <14.688%; conversely, the next discriminator in the right node of hs-cTnI was neutrophil count at a cutoff value of less than 4.870×10^9^/L. Subsequently, the patients with HF were stratified by these branch points: high risk (hs-cTnI <0.068 μg/L, percentage of lymphocytes <14.688%, and cholinesterase <187.916 U/L); low risk (hs-cTnI <0.068 μg/L, percentage of lymphocytes ≥14.688%, and urea <10.113 mmol/L); intermediate risk 1 (hs-cTnI ≥0.068 μg/L, neutrophil count ≥4.870×10^9^/L, and AST <29.003 U/L); and intermediate risk 2 (hs-cTnI ≥0.068 μg/L, neutrophil count <4.870×10^9^/L, and respiratory rate <18.003 breaths/min).

## Discussion

### Principal Findings

In our study, the EHR data–driven prognostic models based on 5 different ML algorithms were developed for predicting 1-year in-hospital mortality, use of positive inotropic agents, and all-cause readmissions within 1 year for patients with HF in a single Chinese class A tertiary comprehensive hospital. These ML models produced better predictive values for prognostic outcomes by growing interpretability than traditional linear methods. Besides, the novel ML techniques could take advantage of a large scale of complex, high-dimensional variables to widen the scope of HF predictive indicators concerning prognostic outcomes.

An integrated EHR system comprises various data resources, including patients’ demographic data, diagnostic information, laboratory test results, and prescriptions. However, many experts revealed that the availability of EHR-derived data is a prerequisite for promoting real-world studies [31,32]. In our study, the structured EHR data from over 10,000 patients in different years can be used directly for analysis without bias of data collection and clinical definitions. A total of 79 variables were applied from EHRs, including demographic information (n=4), current and previous disease history (n=8), HF diagnosis (n=3), vital signs (n=5), laboratory measurements (n=48), interventions (n=4), and medications (n=7). In general, the final target of using rich data derived from mature EHR systems is also patient outcome predictions. With the advancement of data science, different kinds of ML techniques have been broadly employed for model training because of their deep data processing and decision-making capabilities [33]. Compared with previous studies using the traditional LR method [11,20], our results provide better discrimination of risk for prognostic outcomes in a population of patients hospitalized for HF by using ML approaches. The strength of the algorithm could establish complex models and make accurate decisions in relevant big data sets. Besides, far more variables were allowed for modeling by using ML algorithms. Given the 5 methods in the present study, LR was considered the commonly used method in various fields of medicine [12,18]. The RF algorithm involves multiple decision tree creations that identify important predictive features with better accuracy in processing large numbers of highly nonlinear data [20,34]. ANN is a complex network method connected by a large number of simple neurons and simulates the human brain in parallel processing and nonlinear transformation [35]. The SVM approach is designed to build a good classifier using a nonlinear decision boundary between classes of variables that enables the labels from one or more feature vectors [36]. From the perspective of AUC values, it is important to mention that XGBoost performed better than the other 4 methods in predicting 1-year in-hospital mortality, use of positive inotropic agents, and 1-year all-cause readmission in patients with HF. The method’s dominant advantages are its ability to deal with missing values and integrate the power of weaker classifiers by creating combined and weighted variables. Relevant parameters were set to particular values.

Several traditional prognostic models have been established to estimate mortality in hospitalized patients with HF. Our results are consistent with the previous studies of 1-year mortality prediction while demonstrating a better predictive capability. Our study, which predicted outcomes for 13,602 patients with HF using 79 predictive variables, indicated that the ML approaches are superior to the EFFECT method (AUC 0.77) in predicting mortality, with an average AUC of 0.81 [10]. The classic SHFM study showed mortality prediction results within 1 year with AUCs of 0.729-0.76 [37], although the AUC value can be enhanced to 0.78 by combining the SHFM with the BNP level [38]. Furthermore, variables known to be associated with mortality in patients with HF were demonstrated in various previous models. Age, SBP, and level of blood urea nitrogen (BUN) were confirmed as the strongest independent predictors by the EFFECT model [10]. In the landmark Randomized Evaluation of Mechanical Assistance for the Treatment of Congestive Heart Failure (REMATCH) trial model [39], platelet counts, albumin, INR, and AST were associated with 1-year mortality in the HF population with implantable devices. The New York Heart Association class; use of beta-blockers, ACEIs, or ARBs; and presence of heart valve disease or atrial fibrillation remarkably influenced all-cause mortality in the Cardiac and Comorbid Conditions Heart Failure (3C-HF) score [40]. Our study obtained similar findings, demonstrating that in addition to specific biomarkers of HF or myocardial damage, renal function, coagulation indicators, and inflammation indicators are also critical factors regarding prognosis. Therefore, perhaps adopting a large scale of EHR-derived data could enhance discrimination and the predictive range of prognostic outcomes.

Readmission rate is a common index used to assess the quality of health care services for patient populations. Currently, most hospitals and institutions still implement traditional readmission risk models and certain variables to infer readmission probability [41]. Like previous prediction models, we found that the accuracy of mortality prediction was moderate, but the model was relatively poor at predicting readmission rates. Golas et al [42] suggested that the overall 30-day readmission rate was not improved by various ML algorithms, with AUC values around 0.66. Even compared with the established models for HF readmission (AUCs 0.6-0.7), our best ML model is still encouraging [43]. The variables that influenced mortality in the ML models were different from those of readmission among patients with HF. We believe that conventional diagnostic biomarkers (BNP) and vital signs (respiration rate and temperature) could dominate readmission prediction. However, diabetes, coronary heart disease, and gender were the top 3 significant features for predicting readmission using the SVM method. At an interpretable level, a high model AUC does not necessarily indicate that it best fits the scenario.

Positive inotropic agents are a kind of drug that can increase myocardial contractility and cardiac output and are often used to treat patients with HF. Among the drugs included in our study, dopamine and dobutamine have mainly inotropic effects, whereas milrinone, lanatoside C, and levosimendan have extra vasodilation functions [44]. However, they have been gradually limited because of poorer outcomes of patients after accepting inotropic therapy. Thus, controversy still exists regarding their reasonable use in clinical practice. Our study was found to have better performance than two other prognostic models in predicting the use of positive inotropic agents (AUCs in the range of 0.87 to 0.96). Furthermore, this study’s findings are consistent with Aljundi et al [45], who reported that the conventional cardiac biomarker BNP was predictive of inotropic agent use. Many studies have confirmed that comorbidities—such as dyslipidemia, chronic renal and liver impairment, and hyperglycemia—are associated with a higher likelihood of accepting inotropic treatment. Other critically associated variables (INR, AST, and basophil count) were obtained from our models and showed consistent results with common clinical thought. Our models provided good discrimination among patients at high risk for mortality, use of positive inotropic agents, and readmission. Incorporating more variables based on traditional models is of great significance to public health transformation for early identification and individualized intervention of people at high risk of HF outcomes.

The study’s strength was that we also developed a stratified risk assessment tool for the prediction of 1-year in-hospital mortality using the XGBoost algorithm. Far more features—including hs-cTnl, percentage of lymphocytes, percentage of neutrophils, cholinesterase, urea, respiratory rate, and AST—were identified for the first time using an ML approach for mortality prediction. Neutrophilia was reported to be associated with an increased incidence of acute decompensated HF (ADHF) in patients with acute myocardial infarction, and lymphopenia is related to poor prognosis in patients with HF [46,47]. Results from Seo et al [48] showed that cholinesterase was a simple marker for predicting adverse outcomes in patients with ADHF and tended to provide more accurate prognostic information than other objective nutritional features. These results can be attributed to the dimensionality and breadth of EHR-based data, facilitating the real-world study of HF in risk stratification, decision making, and disease management from multiple perspectives. Various HF risk models for predicting mortality have been developed abroad. The ADHERE risk tree by regression analysis [12] has demonstrated that patients with ADHF at low, intermediate, and high risk for in-hospital mortality can be easily identified using BUN, SBP, and creatinine obtained on hospital admission. The MUSIC risk model [49] showed that risk markers including atrial fibrillation, hyponatremia ≤138 mEq/L, N-terminal pro-brain natriuretic peptide >1.000 ng/L, and troponin positive were associated with cardiac mortality in a real-life setting. However, a limitation of the previous model is that it was based on a specific population of patients with HF (ie, symptomatic chronic HF). It should be noted that the etiology, clinical characteristics, and treatments of different phenotypes of HF are quite distinct. Therefore, we compiled all types of patients with HF in this study; it follows that the risk factors predicting mortality are likely to have been more comprehensive and provide new insights for further studies in specific subgroups. These markers possibly provide a more accurate risk evaluation of patients with HF, allowing early implementation of the appropriate intervention in daily public health practice, which leads to better outcomes in patients with HF.

There are several limitations to our study that are worth mentioning. First, this was an in-hospital outcome prediction study based on retrospective use of EHR-derived data. Although our models’ performances were considerable on their own, the predictive power could be further adjusted and compared with the established reference tools. Second, the number of patients who died was small compared with the number of surviving subjects; although rich in terms of clinical variables, the imbalance problem remained. Third, validation in an external cohort was not done in the present study but is planned for subsequent analysis. Fourth, different phenotypes of patients with HF should be taken into account in further model developments.

### Conclusion

EHR-driven models, using novel ML algorithms, were developed to predict 1-year in-hospital mortality, use of positive inotropic agents, and 1-year all-cause readmission in patients hospitalized with HF. The discrimination and performance of our models also outperformed the existing tools constructed using traditional techniques. Besides, identifying a greater range of variables can further improve decisions regarding risk assessment for patients with HF.

## Appendix

Multimedia Appendix 1 Features overview of the complete data set.

## Abbreviations

ACEI: angiotensin-converting enzyme inhibitor
ADHERE: Acute Decompensated Heart Failure National Registry
ADHF: acute decompensated heart failure
ANN: artificial neural network
ARB: aldosterone receptor antagonist
AST: aspartate aminotransferase
AUC: area under the curve
BNP: B-type natriuretic peptide
BUN: blood urea nitrogen
EFFECT: Enhanced Feedback for Effective Cardiac Treatment
EHR: electronic health record
HF: heart failure
hs-cTnI: high-sensitivity cardiac troponin I
ICD-10: International Classification of Diseases, 10th Revision
INR: international normalized ratio
LR: logistic regression
ML: machine learning
MLP: multilayer perceptron
REMATCH: Randomized Evaluation of Mechanical Assistance for the Treatment of Congestive Heart Failure
RF: random forest
ROC: receiver operating characteristic
SBP: systolic blood pressure
SHAP: Shapley additive explanation
SHFM: Seattle Heart Failure Model
SVM: support vector machine
XGBoost: extreme gradient boosting
