# Supplementary material for: Machine Learning–Driven Models to Predict Prognostic Outcomes in Patients Hospitalized With Heart Failure Using Electronic Health Records: Retrospective Study
Source: J Med Internet Res. 2021 Apr 19;23(4):e24996. doi: 10.2196/24996 (PMC8094022; doi:10.2196/24996)
Supplement: Multimedia Appendix 1 [file jmir_v23i4e24996_app1.docx]

**S1 Table.** **Features overview of the complete data set.**

| **Variables** |  | **Data filling rates (%)** |  | **Mean ± SD /Counts（%）** |
| --- | --- | --- | --- | --- |
| **Total number of participants** |  | 13602 |  |  |
| **Demographic data** |  |  |  |  |
| Age（years） |  | 99.99 |  | 72.0 (63.0-80.0) |
| Gender |  | 100 |  |  |
| Male |  |  |  | 7142 (52.5) |
| Female |  |  |  | 6460 (47.5) |
| Smoking history（n/%） |  | 95.87 |  |  |
| Yes |  |  |  | 3397 (25.0) |
| No |  |  |  | 9643(70.9) |
| Drinking history（n/%） |  | 92.74 |  |  |
| Yes |  |  |  | 1884 (13.9) |
| No |  |  |  | 10730 (78.9) |
| **Comorbidities（Yes）** |  |  |  |  |
| Diabetes mellitus |  | 100 |  |  |
| Yes |  |  |  | 3795 (27.9) |
| No |  |  |  | 9807 (72.1) |
| Hypertension |  | 100 |  |  |
| Yes |  |  |  | 7411 (54.5) |
| No |  |  |  | 6191 (45.5) |
| Dyslipidemia (hyperlipemia) |  | 100 |  |  |
| Yes |  |  |  | 8762 (64.4) |
| No |  |  |  | 4840 (35.9) |
| COPD |  | 100 |  |  |
| Yes |  |  |  | 53 (0.4) |
| No |  |  |  | 13549 (99.6) |
| Chronic renal disease |  | 100 |  |  |
| Yes |  |  |  | 157 (1.2) |
| No |  |  |  | 13445 (98.8) |
| Tumors |  | 100 |  |  |
| Yes |  |  |  | 580 (4.3) |
| No |  |  |  | 13022 (95.7) |
| **The etiology of HF** |  |  |  |  |
| Coronary heart disease |  | 100 |  |  |
| Yes |  |  |  | 8159 (60.0) |
| No |  |  |  | 5443 (40.0) |
| Cardiomyopathy |  | 100 |  |  |
| Yes |  |  |  | 1229 (9.0) |
| No |  |  |  | 12373 (91.0) |
| Valvular heart disease |  | 100 |  |  |
| Yes |  |  |  | 2480 (18.2) |
| No |  |  |  | 11122 (81.8) |
| Cardiac arrhythmia; |  | 100 |  |  |
| Yes |  |  |  | 5973 (43.9) |
| No |  |  |  | 7629 (56.1) |
| Cardiovascular surgery history |  | 100 |  |  |
| Yes |  |  |  | 2634 (19.4) |
| No |  |  |  | 10968 (80.6) |
| **Vital signs** |  |  |  |  |
| Blood pressure（mmHg） |  |  |  |  |
| DBP |  | 96.40 |  | 70.0 (80.0-90.0) |
| SBP |  | 96.43 |  | 120.0 (139.0-152.0) |
| Heart rate（Times/min） |  | 97.18 |  | 68.0 (77.0-90.0) |
| Respiratory rate（Times/min） |  | 97.02 |  | 17.0 (18.0-19.0) |
| Temperature |  | 93.30 |  | 36.2 (36.0-36.4) |
| NYHA classification（n%） |  | 100 |  | 8014 (58.9) |
| IV |  |  |  | 1537 (19.2) |
| III |  |  |  | 4102 (51.2) |
| II |  |  |  | 1760 (22.0) |
| I |  |  |  | 12 (0.1) |
| None |  |  |  | 5588 (69.7) |
| **Laboratory indicators** |  |  |  |  |
| BNP (pg/ml) |  | 83.57 |  | 341.5 (111.9-895.3) |
| hs-cTnl (ug/L) |  | 81.48 |  | 0.03 (0.01-0.13) |
| CK-MB（ug/L） |  | 84.91 |  | 1.5 (0.8-2.7) |
| HGB（g/L） |  | 94.13 |  | 6.6 (5.4-8.3) |
| PLT (10e9/L) |  | 93.77 |  | 192.0 (155.0-235.0) |
| WBC（x109/L） |  | 93.86 |  | 6.6 (5.3-8.3) |
| RBC (10e12/L) |  | 93.86 |  | 4.3 (3.9-4.7) |
| Lymph (10e9/L) |  | 93.81 |  | 1.6 (1.2-2.1) |
| Neut (10e9/L) |  | 93.80 |  | 3.1 (4.1-5.6) |
| MPV（fL） |  | 92.80 |  | 10.7 (10.0-11.4) |
| Hct (%) |  | 93.43 |  | 38.6 (32.8-42.7) |
| Baso（x109/L） |  | 87.52 |  | 0.02 (0.01-0.03) |
| Mono（x109/L） |  | 93.81 |  | 0.5 (0.4-0.7) |
| Mono% |  | 93.81 |  | 7.9 (6.3-9.7) |
| MCV（fL） |  | 93.87 |  | 91.0 (87.8-94.2) |
| PCT |  | 92.80 |  | 0.2 (0.2-0.3) |
| Neut (%) |  | 93.80 |  | 63.4 (55.8-71.9) |
| Baso (%) |  | 87.98 |  | 0.3 (0.2-0.5) |
| EoS (%) |  | 88.05 |  | 0.1 (0.1-0.2) |
| EoS（x109/L） |  | 85.77 |  | 0.9 (1.7-2.8) |
| Lymph (%) |  | 93.81 |  | 25.2 (17.6-32.4) |
| TBIL（μmol/L） |  | 95.78 |  | 14.5 (10.5-20.6) |
| DBIL（μmol/L） |  | 87.50 |  | 4.6 (3.2-7.0) |
| Glu（mmol/L） |  | 91.34 |  | 5.5 (4.9-6.9) |
| **LPS（U/L）** |  | **5.20** |  | **79.0 (42.0-147.0)** |
| **Cys-C（mg/L）** |  | **67.78** |  | **1.2 (1.0-1.6)** |
| **apoB（g/L）** |  | **24.58** |  | **0.8 (0.7-1.0)** |
| **apoA（g/L）** |  | **24.58** |  | **0.9 (0.8-1.1)** |
| LPa（mg/L） |  | 83.27 |  | 152.2 (83.0-278.0) |
| HDL-C（mmol/L） |  | 90.48 |  | 1.5 (1.0-39.0) |
| LDL-C（mmol/L） |  | 90.48 |  | 3.5 (2.3-90.0) |
| TC（mmol/L） |  | 90.47 |  | 5.8 (4.2-154.0) |
| TG（mmol/L） |  | 90.47 |  | 2.1 (1.1-91.0) |
| ALT（U/L） |  | 95.77 |  | 20.0 (13.0-33.0) |
| AST（U/L） |  | 95.78 |  | 21.0 (16.0-31.0) |
| GGT（U/L） |  | 85.96 |  | 35.0 (22.0-64.0) |
| ALB（g/L） |  | 95.17 |  | 39.2 (36.0-41.8) |
| GLO（g/L） |  | 95.18 |  | 26.8 (23.6-30.4) |
| ALB/GLO |  | 95.16 |  | 1.5 (1.2-1.7) |
| TP（g/L） |  | 95.17 |  | 65.8 (61.4-70.4) |
| Crea（μmol/L） |  | 95.63 |  | 77.0 (63.0-100.0) |
| Na（mmol/L） |  | 96.77 |  | 141.0 (138.0-143.0) |
| K (mmol/L) |  | 96.89 |  | 4.0 (3.7-4.3) |
| Ca（mmol/L） |  | 96.33 |  | 2.2 (2.1-2.3) |
| UA（μmol/L） |  | 89.54 |  | 391.0 (331.0-492.0) |
| Urea（mmol/L） |  | 95.58 |  | 7.2 (5.6-9.7) |
| ALP（U/L） |  | 95.78 |  | 75.0 (62.0-93.0) |
| ChE（U/L） |  | 87.56 |  | 290.0 (226.0-361.0) |
| INR |  | 92.88 |  | 1.1 (1.0-1.2) |
| PT（s） |  | 92.84 |  | 11.9 (11.1-13.3) |
| Fbg（g/L） |  | 88.23 |  | 3.1 (2.5-3.8) |
| APTT（s） |  | 88.29 |  | 26.9 (23.9-31.1) |
| **D-Dimer（ug/L）** |  | **75.47** |  | **600.0 (280.0-1360.0)** |
| **CRP** |  | **9.15** |  | **6.7 (3.3-21.0)** |
| **PCT** |  | **15.13** |  | **0.1 (0.1-0.4)** |
| **HbA1c** |  | **25.59** |  | **6.5 (5.8-7.7)** |
| **ESR** |  | **9.76** |  | **17.0 (7.0-32.0)** |
| **HCY (umol/L)** |  | **50.27** |  | **16.7 (13.1-21.5)** |
| CRT（n/%） |  | 100 |  |  |
| Yes |  |  |  | 44（3.4） |
| No |  |  |  | 13558（99.7） |
| ICD implantation（n/%） |  | 100 |  |  |
| Yes |  |  |  | 32(0.2) |
| No |  |  |  | 13570(99.8) |
| Permanent pacemaker（n/%） |  | 100 |  |  |
| Yes |  |  |  | 353(2.6) |
| No |  |  |  | 13249(97.4) |
| Temporary pacemaker （n/%） |  | 100 |  |  |
| Yes |  | 0.11 |  | 15(0.1) |
| No |  | 99.89 |  | 13587(99.9) |
| **Medication during hospitalization** |  |  |  |  |
| ACEI/ARB（n/%） |  | 100 |  |  |
| Yes |  |  |  | 8174(60.1) |
| No |  |  |  | 5428(39.9) |
| β-blocker（n/%） |  | 100 |  |  |
| Yes |  |  |  | 10607(78.0) |
| No |  |  |  | 2995(22.0) |
| ALD blocker（n/%） |  | 100 |  |  |
| Yes |  |  |  | 8630(63.4) |
| No |  |  |  | 4972(36.6) |
| Statins（n/%） |  | 100 |  |  |
| Yes |  |  |  | 8709(64.0) |
| No |  |  |  | 4893(36.0) |
| Aspirin（n/%） |  | 100 |  |  |
| Yes |  |  |  | 8586(63.1) |
| No |  |  |  | 5016(36.9) |
| Diuretic（n/%） |  | 100 |  |  |
| Yes |  |  |  | 11435(84.1) |
| No |  |  |  | 2167(15.9) |
| Digoxin（n/%） |  | 100 |  |  |
| Yes |  |  |  | 2497(18.4) |
| No |  |  |  | 11105(81.6) |
| **Outcomes** |  |  |  |  |
| **In-hospital deaths** |  |  |  | 537(3.9) |
| **Positive inotropic agents use** |  |  |  | 2779（20.4） |
| Dopamine |  | 100 |  |  |
| Yes |  |  |  | 1726(12.7) |
| No |  |  |  | 11876(87.3) |
| Dobutamine Hydrochloride |  | 100 |  |  |
| Yes |  |  |  | 264(1.9) |
| No |  |  |  | 13338(98.1) |
| Milrinone |  | 100 |  |  |
| Yes |  |  |  | 377(2.8) |
| No |  |  |  | 13225(97.2) |
| Levosimendan |  | 100 |  |  |
| Yes |  |  |  | 44(0.3) |
| No |  |  |  | 13558(99.7) |
| Cedilanid |  | 100 |  |  |
| Yes |  |  |  | 1261(9.3) |
| No |  |  |  | 12341(90.7) |
| **Readmissions** |  |  |  |  |
| 30d |  |  |  | 657(4.8) |
| 180d |  |  |  | 2009(14.8) |
| 1Year |  |  |  | 2878(21.2) |

**NOTE:**

**Highlights:** Variables with missing values greater than 20%.

**COPD：**Chronic obstructive pulmonary disease; **HF:** Heart failure; **DBP:** Diastolic blood pressure; **SBP:** Systolic blood pressure; **NYHA:** New York Heart Association; **BNP:** Type B natriuretic peptide; **CK-MB:** Creatine kinase MB; **HGB:** Hemoglobin; **PLT:** Platelets counts; **WBC:** White blood cell counts; **RBC:** Red blood cell counts; **Lymph:** Lymphocyte counts; **Neut:** Neutrophil counts; **MPV:** Mean platelet volume; **Hct:** Hematocrit; **Baso:** Basophil counts; **Mono:** Monocyte counts; **MCV:** Mean corpuscular volume; **PCT:** Thrombocytocrit; **EoS:** Eosinophil counts; **TBIL:** Total bilirubin**; DBIL:** Direct Bilirubin; **Glu:** Glucose; **LPa:** Lipoprotein a; **HDLC:** High density lipoprotein; **LDLC:** Low Density Lipoprotein; **TC:** Total cholesterol; **TG:** Triglyceride; **ALT:** Alanine transaminase; **AST:** Aspartate transaminase; **GGT:** Gamma-glutamyl transpeptidase; **ALB:** Albumin; **GLO:** Globulin; **TP:** Total protein; **Crea:** Creatinine; **UA:** Uric Acid; **ALP:**A Lkaline Phosphatase; **ChE:** Cholinesterase; **INR:** International Normalized Ratio; **PT:** Prothrombin time; **Fbg:** Fibrinogen; **APTT:** Activated partial thromboplastin time; **CRP:** C-reactive protein; **PCT:** Procalcitonin; **HbA1c:** Glycosylated hemoglobin; **ESR:** End-stage renal; **HCY:** Homocysteine; **CRT:** Cardiac resynchronisation Therapy; **ICD:** Implantable cardiac defibrillator; **ACEI:** Angiotensin-Converting Enzyme Inhibitors; **ARB:** Angiotensin Receptor Blockers.
